# Short- and long-term outcomes in ypT2 rectal cancer patients after neoadjuvant therapy and local excision: a multicentre observational study

**DOI:** 10.1007/s10151-022-02712-y

**Published:** 2022-10-14

**Authors:** Roberto Peltrini, Simone Castiglioni, Nicola Imperatore, Monica Ortenzi, Daniela Rega, Valentina Romeo, Valerio Caracino, Edoardo Liberatore, Massimo Basti, Emanuele Santoro, Umberto Bracale, Paolo Delrio, Felice Mucilli, Mario Guerrieri, Francesco Corcione

**Affiliations:** 1 Department of Public Health, School of Medicine and Surgery, University of Naples Federico II, Via Pansini 5, 80131 Naples, Italy; 2 Department of Medical, Oral and Biotechnological Sciences, University G. D’Annunzio Chieti-Pescara, Chieti, Italy; 3 Gastroenterology and Endoscopy Unit, AORN Antonio Cardarelli, Naples, Italy; 4 Department of General and Emergency Surgery, Marche Polytechnic University, Ancona, Italy; 5 Colorectal Surgical Oncology, Department of Abdominal Oncology, Istituto Nazionale Tumori-IRCCS “Fondazione G. Pascale”, Naples, Italy; 6 Department of Surgery, San Giovanni Addolorata Hospital Complex, Rome, Italy; 7 General and Emergency Surgery Unit, Santo Spirito Hospital, ASL Pescara, Pescara, Italy; 8 General Surgery Unit, “San Liberatore” Hospital, Atri, ASL Teramo, Teramo, Italy

**Keywords:** Local excision, Chemoradiotherapy, Rectal cancer, Transanal endoscopic microsurgery, Transanal minimally invasive surgery, Organ preservation

## Abstract

**Background:**

Although local excision (LE) after neoadjuvant treatment (NT) has achieved encouraging oncological outcomes in selected patients, radical surgery still remains the rule when unfavorable pathology occurs. However, there is a risk of undertreating patients not eligible for radical surgery. The aim of this study was to evaluate the outcomes of patients with pathological incomplete response (ypT2) in a multicentre cohort of patients undergoing LE after NT and to compare them with ypT0-is-1 rectal cancers.

**Methods:**

From 2010 to 2019, all patients who underwent LE after NT for rectal cancer were identified from five institutional retrospective databases. After excluding 12 patients with ypT3 tumors, patients with ypT2 tumors were compared to patients with ypT0-is-1 tumors). The endpoints of the study were early postoperative and long-term oncological outcomes.

**Results:**

A total of 177 patients (132 males, 45 females, median age 70 [IQR 16] years) underwent LE following NT**.** There were 46 ypT2 patients (39 males, 7 females, median age 72 [IQR 18.25] years) and 119 ypT0-is-1 patients (83 males, 36 females, median age 69 [IQR 15] years). Patients with pathological incomplete response (ypT2) were frailer than the ypT0-is-1 patients (mean Charlson Comorbidity Index 6.15 ± 2.43 vs. 5.29 ± 1.99; *p* = 0.02) and there was a significant difference in the type of NT used for the two groups (long- course radiotherapy: 100 (84%) vs. 23 (63%), *p* = 0.006; short-course radiotherapy: 19 (16%) vs. 17 (37%), *p* = 0.006). The postoperative rectal bleeding rate (13% vs. 1.7%; *p* = 0.008), readmission rate (10.9% vs. 0.8%; *p* = 0.008) and R1 resection rate (8.7% vs. 0; *p* = 0.008) was significantly higher in the ypT2 group. Recurrence rates were comparable between groups (5% vs. 13%; *p* = 0.15). Five-year overall survival was 91.3% and 94.9% in the ypT2 and ypT0-is-1 groups, respectively (*p* = 0.39), while 5-year cancer specific survival was 93.4% in the ypT2 group and 94.9% in the ypT0-is-1 group (*p* = 0.70). No difference was found in terms of 5-year local recurrence free-survival (*p* = 0.18) and 5-year distant recurrence free-survival (*p* = 0.37).

**Conclusions:**

Patients with ypT2 tumors after NT and LE have a higher risk of late-onset rectal bleeding and positive resection margins than patients with complete or near complete response. However, long-term recurrence rates and survival seem comparable.

## Introduction

Total mesorectal excision (TME) is the cornerstone for the surgical treatment of rectal cancer [1–3]. In order to improve local control of disease, neoadjuvant chemoradiotherapy has become the standard for locally advanced low-medium rectal tumors (T3-4 and/or N+) as it significantly reduces the rate of local recurrence compared to both adjuvant therapy and optimal surgery alone [4–6]. However, postoperative complications occur up to 50% of cases [7–9] with a significant negative impact on quality of life, bowel, urinary and sexual function, often including the need for a permanent stoma [10–15].

Local excision (LE) after neoadjuvant treatment (NT) is considered an organ preservation strategy that has demonstrated satisfying oncological and functional outcomes [16–22]. In particular, the local recurrence rate is less than 5% in patients with a pathologic major response (ypT0 and ypT1) and more than 95% do not have an ostomy with a preserved rectum [23]. Although some evidence suggests that LE after NT may be considered safe and effective in selected patients with complete or near complete pathologic response (ypT0-1), conservative management is usually not adequate for residual cancer invading the muscularis propria (ypT2) and a completion TME is required. However, completion TME after LE is performed in less than 30% of cases, resulting in a risk of undertreatment for many patients [24]. Despite unfavorable pathology, some patients may be considered not eligible for major surgery due to severe comorbidities, and others may refuse a permanent colostomy when scheduled for abdominoperineal resection (APR).

Therefore, the aim of this study was to evaluate clinical outcomes of patients with unfavorable pathology (ypT2). Considering a full-thickness excision up to perirectal fat and a safe margin of 1 cm from the lesion as conservative treatment, the residual tumor within the bowel wall is ideally removed in ypT2 cancers. Although a 20% of risk of positive mesorectal lymph nodes in ypT2 patients was reported [25, 26], we hypothesized that their long-term outcomes were similar to those of ypT0-1 patients. If confirmed, conservative treatment may have a role even in ypT2 patients who may benefit from an organ-preservation strategy.

## Materials and methods

From 2010 to 2019, all patients who underwent LE after NT for rectal cancer were identified from five institutional retrospective databases. Two of five centers were considered high-volume centers for rectal cancer surgery and they were experienced in organ preservation. Preoperative workup included medical history, physical examination with digital rectal examination, colonoscopy with biopsy to histologically confirm rectal cancer. Pelvic magnetic resonance imaging (MRI) and endoscopic ultrasound were performed for radiological evaluation before NT. Computer tomography (CT) scan was performed to detect distant metastases. Long-course radiotherapy (RT) or short-course RT was administered based on tumor stage. Fluoropyrimidine-based chemotherapy was administered concomitantly with RT at a total dose of 50.4 Gy given in 28 fractions of 1.8 Gy each (long course RT). Short course RT consisted of 5 Gy daily for 5 days without chemotherapy. Re-evaluation of patients after preoperative therapy was performed by clinical examination using digital rectal examination, proctoscopy, MRI and ultrasound as imaging 8 weeks after NT.

Surgical strategy was discussed by a multidisciplinary team and with patients. Transanal LE was chosen as treatment in the cases of complete clinical response and for any reason including patients deemed not eligible for major surgery, who refused radical surgery or who refused permanent colostomy when APR was required. APR was indicated for tumors that involved the anal sphincter or the levator muscles and in cases of unacceptable sphincter function. Complete clinical response was defined as the absence of any irregularity, mass, ulceration, or stenosis (including metastatic lymph nodes) during clinical rectal examination or the presence of endoluminal scars and telangiectasias. Informed consent was obtained from all patients.

Full-thickness rectal wall excision was performed by transanal endoscopy microsurgery (TEM) or transanal minimally invasive surgery (TAMIS) platforms or by conventional transanal excision (TAE) between 8 and 12 weeks after NT. The rectal wall defect was left open or sutured according to the surgeon's preference.

Demographic and preoperative clinical characteristics (age, sex, body mass index (BMI), Charlson comorbidity index (CCI), American Society of Anesthesiologists (ASA) class, NT type, tumor location), surgical and pathological features (transanal procedure, pTNM classification, margin involvement), postoperative complication and readmission rates associated with long-term oncologic outcomes, were obtained from the database of each hospital. Subsequently, all data were anonymized and recorded in a single comprehensive database.

Local recurrence was defined as endoluminal recurrent disease or within mesorectal fat. In cases of suspicion, a pelvic MRI and an endoscopic assessment were performed. A pathological diagnosis was obtained by biopsy. Distant recurrence included liver or lung metastases or nodal metastasis beyond the regional nodes. In these cases, imaging was always performed.

The pathology report included complete responses (ypT0) defined as absence of viable tumor cells detected in the surgical specimen or near complete/incomplete response (ypTis-1/ypT2-3) based on residual tumor infiltration. Patients were followed up every 3–6 months for the first 2 years after surgery and then every 6 months for a total of 5 years. CT scans of the chest, abdomen, and pelvis; serum markers; and colonoscopy were performed according to the guidelines.

Outcomes of ypT2 patients for whom LE after NT was not considered curative and completion TME required were compared with outcome of ypT0-is-1 patients. This study was reported according to the Strengthening the Reporting of Observational Studies in Epidemiology (STROBE) guidelines [27].

### Statistical analysis

Data were analysed using the Statistical Package for Social Sciences (SPSS software v.15.0, Chicago IL,USA) for Windows and StatsDirect statistical software (version 3.0). The descriptive statistics used included determination of mean values and standard deviation (SD) of the continuous variables, and of percentages and proportions of the categorical variables. Statistical analysis was performed using X2, Student’s *t* test and Mann–Whitney *U* test, when indicated. The probability of 5-year survival for each group (ypT0-Tis-T1 vs. ypT2) was also calculated using the Kaplan–Meier method and the curves obtained were compared using the log-rank. Results were considered statistically significant when *p* < 0.05.

## Results

From 2010 to 2019 a total of 177 patients (132 males, 45 females, median age 70 [IQR 16] years) underwent LE following NT. There were 46 ypT2 patients (39 males, 7 females, median age 72 [IQR 18.25] years) and 119 ypT0-is-1 patients (83 males, 36 females, median age 69 [IQR 15] years). Associated comorbidities resulted in a mean CCI of 5.29 (± 1.99) and 68 patients (38.4%) were considered ASA 3–4. Cancer was localized in the lower or middle rectum in 170 patients (96%) (up to 10 cm from the anal verge). Preoperative stage according to the TNM staging system was as follows: T1 in 6 (3.4%), T2 in 50 (28.2%), T3 in 117 (66.1%), T4 in 4 (2.3%) and N+ in 70 (39.5%) patients. Most patients received long-course RT (140 patients, 79.1%) rather than short-course RT (37 patients, 20.9%). LE was performed using a minimally invasive approach in 106 (59.9%) (TEM) and 46 (26%) (TAMIS) patients, conventional TAE in the remaining 25 (14.1%). After surgery, a pathological complete or near complete response (ypT0-is-1) was achieved in 119 patients (67.2%), ypT2 in 46 (26%) and ypT3 in 12 (6.7%).

### Comparative outcomes of ypT0-is-1 vs. ypT2

After excluding 12 ypT3 tumors, the remaining 46 patients with incomplete pathological response (ypT2) were compared with 119 ypT0-is-1 patients. As regards baseline characteristics and interventions, the ypT2 group had a higher CCI (*p* = 0.02). Additionally, 100 (84%) and 29 patients (63%) received long course RT in ypT0-is-1 and ypT2 group, respectively (*p* = 0.006); while short course RT was used significantly less often in the ypT0-is-1 group than in the ypT2 group (16% vs. 37%; *p* = 0.006) (Table 1).

**Table 1:** Baseline patients and tumor characteristics

	ypT0-Tis-T1 *n* = 119 (%)	ypT2 *n* = 46 (%)	*p* value
*Sex*			
M	83 (69.7)	39 (84.8%)	0.07
F	36 (30.3)	7 (15.2%)	0.07
*Age, years, median (IQR)*	69 (15)	72 (18.25)	0.24
*BMI (kg/m*^*2*^*) mean ± SD*	25.31 ± 3.75	24.99 ± 3.72	0.62
*ASA class*			
1–2	78 (65.5)	23 (50)	0.09
3–4	41 (34.5)	23 (50)	0.09
*Charlson Comorbidity Index*	5.29 ± 1.99	6.15 ± 2.43	0.02
*Distance from anal verge (cm)*			
Mean ± SD	6.15 ± 2.74	6.16 ± 2.22	0.98
< 5 cm	32 (26.9)	9 (19.6)	0.43
5–10 cm	80 (67.2)	37 (80.4)	0.13
> 10 cm	7 (5.9)	0 (0)	0.2
*c Stage*			
T1	5 (4.2)	1 (2.2)	0.87
T2	36 (30.3)	11 (23.9)	0.53
T3	77 (64.7)	31 (67.4)	0.88
T4	1 (0.8)	3 (6.5)	0.12
N+	45 (37.8)	22 (47.8)	0.32
*Neoadjuvant treatment*			
Long course RT	100 (84)	29 (63)	0.006
Short course RT	19 (16)	17 (37)	0.006

*BMI* body mass index, *ASA* American Society of Anesthesiologists, *IQR* interquartile range

There was a significantly higher postoperative bleeding rate in the ypT2 group (13% vs. 1.7%, *p* = 0.008). In contrast, overall and specific postoperative complication rates in terms suture line dehiscence, and rectal abscess were similar between the two groups, as was length of hospital stay (*p* > 0.05). Moreover, the ypT2 group had a higher readmission rate (10.9% vs. 0.8%; *p* = 0.008), due to rectal bleeding in all cases (Table 2).

**Table 2:** Postoperative outcomes

	ypT0-Tis-T1 *n* = 119 (%)	ypT2 *n* = 46 (%)	*p* value
*Postoperative complications*			
Bleeding	2 (1.7)	6 (13)	0.008
Suture line dehiscence	4 (3.4)	2 (4.3)	0.87
Rectal abscess	5 (4.2)	1 (2.2)	0.87
Overall complications	11 (9.2)	9 (10.9)	0.12
*Length of hospital stay (days), mean + SD*	4.19 ± 2.68	4.11 ± 2.79	0.86
*30-day readmission*	1 (0.8)	5 (10.9)	0.008
*Resection margin*			
R1	0 (0)	4 (8.7)	0.008
*Relapse*			
Overall patients with relapse	6 (5)	4 (8.7)	0.6
Overall (local + systemic)	6 (5)	6 (13)	0.15
Local	4 (3.4)	4 (8.7)	0.30
Systemic	2 (1.7)	2 (4.3)	0.66
*Salvage TME*	3 (2.5)	3 (6.5)	0.44
*Overall mortality rate*	6 (5)	5 (10.9)	0.31
*Length of follow-up (months) Mean ± SD*	71.29 ± 34.52	61.56 ± 41.86	0.13

*TME* total mesorectal excision

Although resection margin after LE was involved (R1) in 8.7% of cases in ypT2 patients and never in the comparative group (*p* = 0.008), no difference in local or distant recurrence and salvage TME rate was reported (local recurrence: 3.4% vs. 8.7%; *p* = 0.30—distant recurrence: 1.7% vs. 4.3%; *p* = 0.66). Three ypT2 patients who did not have local recurrence underwent TME after LE.

**Fig. 1:**
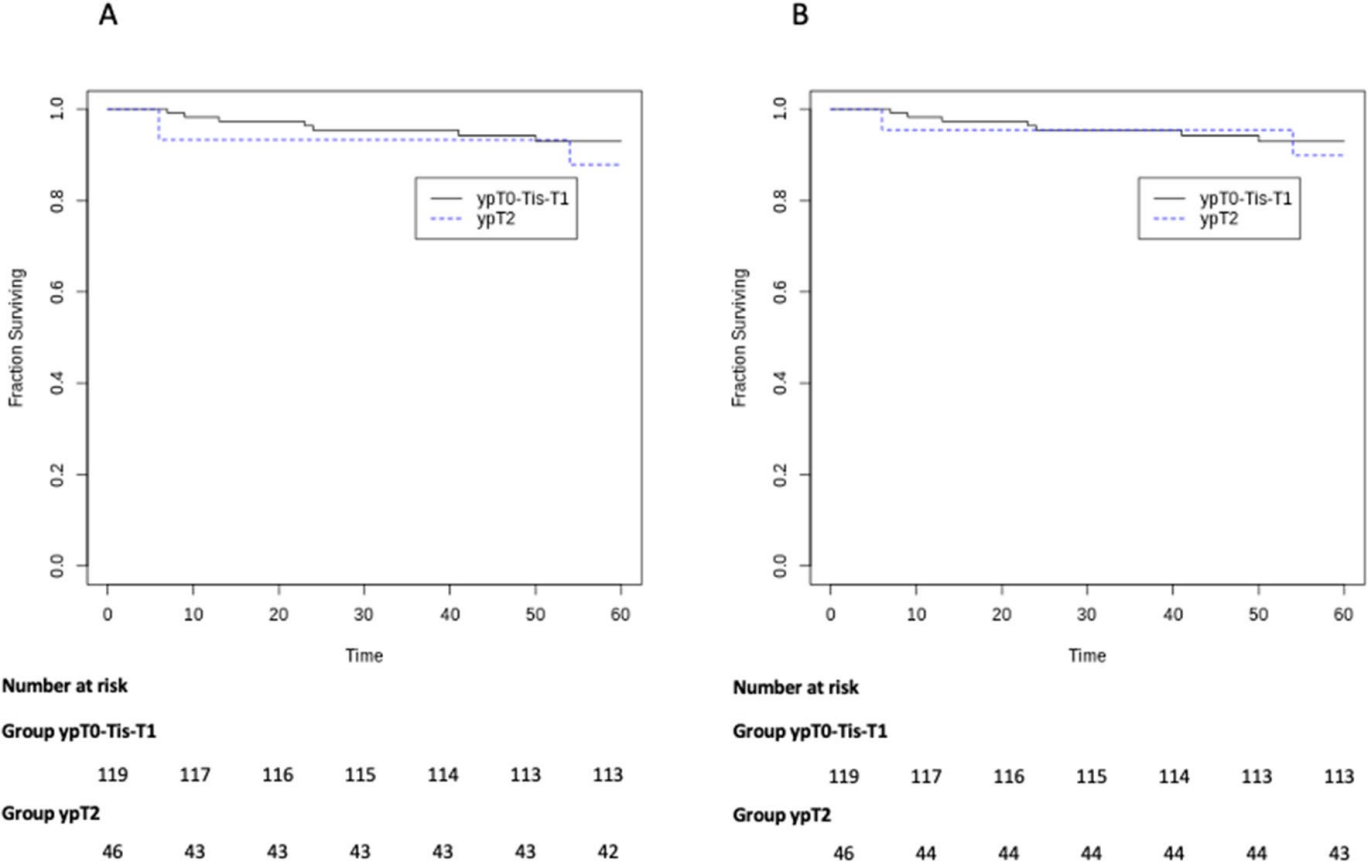
**a** Kaplan–Meier curve for 5-year survival **b** Kaplan–Meier curve for 5-year cancer-specific survival

**Fig. 2:**
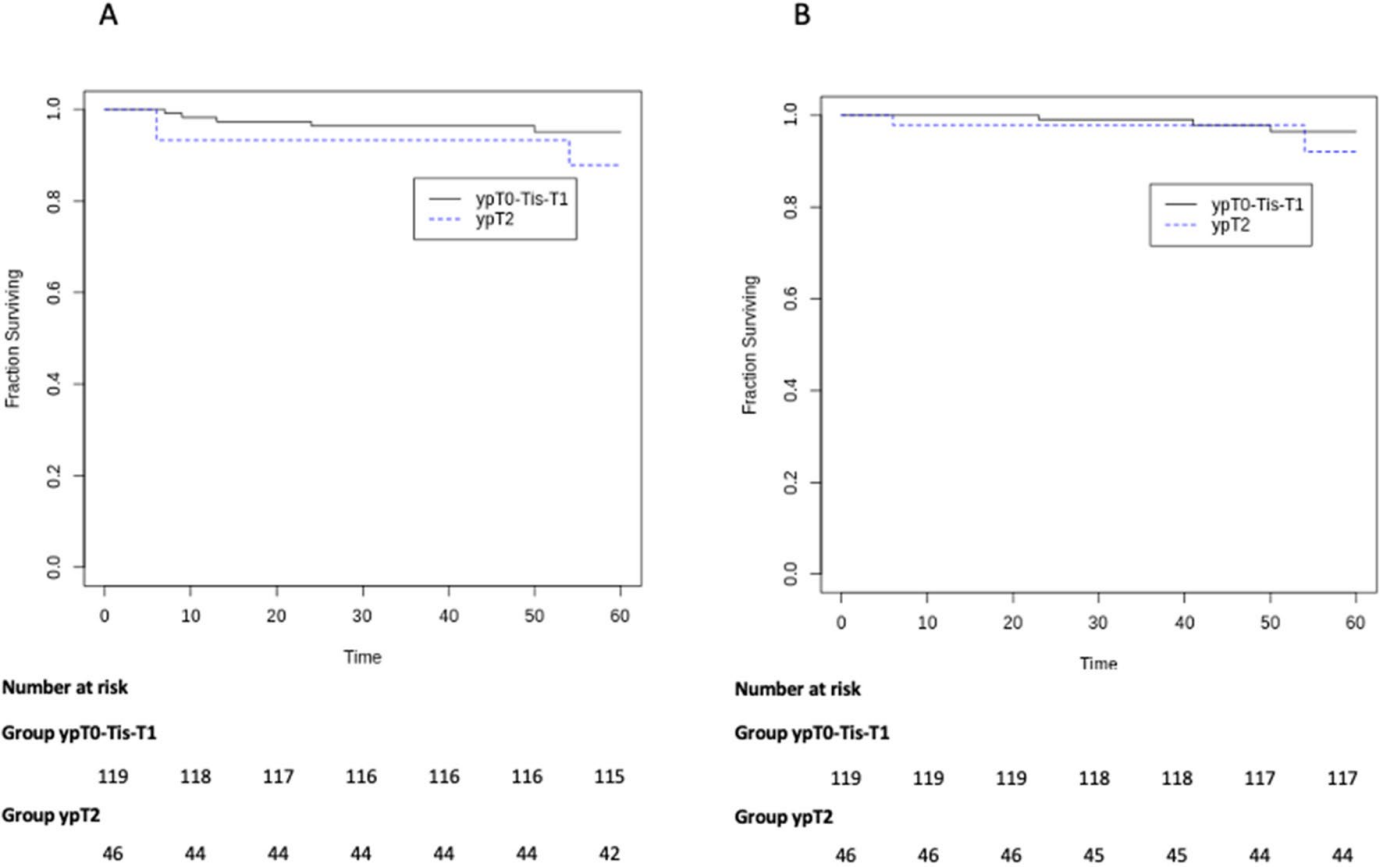
**a** Kaplan–Meier curve for 5-year local recurrence free-survival **b** Kaplan–Meier curve for 5-year distant recurrence free-survival

Overall, ten patients had recurrences. Of these, two patients had both local recurrence and distant recurrence in the ypT2 group. The mean time to recurrence was 19.5 ± 12.67 months in ypT0-Tis-T1 group vs. 18 ± 24 months in ypT2 group (*p* = 0.89). Characteristics of patients developing tumor relapse are shown in Table 3. At the time of follow- up, 5-year overall survival was 94.9% and 91.3% in the ypT0-is-1 and ypT2 group, respectively (*p* = 0.39) (Fig. 1A), while 5-year cancer specific survival was 94.9% and 93.4% in the ypT0-is-1 and ypT2 group, respectively (*p* = 0.70) (Fig. 1B). Moreover, the 5-year local recurrence free-survival was 96.6% and 91.3% in the ypT0-is-1 and 91.3% in the ypT2 group (*p* = 0.18) (Fig. 2A), while 5-year distant recurrence free-survival was 98.3% in the ypT0-is-1 and 95.6% in the ypT2 group (*p* = 0.37) (Fig. 2B).

**Table 3:** Characteristics of patients developing tumor recurrence

	Type of relapse	Time to recurrence (months)	cTN	Distance from a.v. (cm)	Neoadjuvant treatment	Transanal surgery	ypT	Resection margin status	Salvage TME	Follow up after LE (months)	Survival status	Cause of death
Patient 1	DR	23	T3N0	7	SCRT	TAMIS	ypT1	R0	No	50	Death	Cancer-related
Patient 2	LR	9	T2N0	2	LCRT	TAMIS	ypT0	R0	APR	12	Death	Cancer-related
Patient 3	LR	24	T2N0	5	LCRT	TEM	ypT1	R0	No^a^	52	Death	Cancer-related
Patient 4	DR + LR	54	T3N+	6	LCRT	TAE	ypT2	R0	APR	54	Death	Cancer-related
Patient 5	DR + LR	6	T2N0	5	SCRT	TAMIS	ypT2	R0	LAR	63	Death	Cancer-related
Patient 6	LR	6	T3N+	10	SCRT	TAMIS	ypT2	R0	No	7	Death	Not Cancer-related
Patient 7	LR	6	T4N+	2	LCRT	TAE	ypT2	R0	APR	24	Death	Cancer-related
Patient 8	DR	41	T3N+	5	LCRT	TAMIS	ypTis	R0	No	44	Death	Cancer-related
Patient 9	LR	7	T3N+	1	LCRT	TAE	ypT1	R0	LAR	13	Alive	–
Patient 10	LR	13	T3N+	3.5	LCRT	TAE	ypTis	R0	APR	26	Alive	–

*a.v.* anal verge, *TME* total mesorectal excision, *LR* local recurrence, *DR* distant recurrence, *APR* abdominoperineal resection, *LAR* low anterior resection

^a^Colostomy

## Discussion

We investigated differences between ypT0-is-1 and ypT2 rectal tumors after NT and LE from a multicenter cohort of patients to assess early postoperative and long-term oncological outcomes. Patients with pathological incomplete response (ypT2) were frailer and more received short course RT than the ypT0-is-1 group. Postoperative rectal bleeding, readmission rate and R1 resection rate were significantly higher in the ypT2 group. Despite unfavorable pathology after NT and LE, no significant differences in long-term oncological outcomes between ypT0-is-1 and ypT2 patients were observed.

Patients with adverse pathology following NT and LE usually undergo completion TME. However, a significant number of patients fail to undergo this completion surgery [20]. Data from Dutch Colorectal Audit (DCRA) [24] showed more than 50% of all patients who underwent LE for rectal cancer had an indication for completion TME based on histopathologic characteristics but completion TME was performed in only around one third of this group. Even considering pT ≥ 2 stage after LE alone, 62% of patients failed to undergo completion TME. When we investigated the outcomes of this group of potentially undertreated patients we observed that the use of short course RT was significantly greater in the ypT2 group (*p* = 0.006), and the use of long-course RT was significantly greater in the ypT0-is-1 group (*p* = 0.006). This is in line with a major downstaging effect of long course RT. In fact, in the TROG 01.04 trial [28] 15% of patients had a pathologic complete response (ypT0) after long-course RT compared with 1% following short-course RT. Similarly, in a Polish trial [29], comparing NT (50·4 Gy plus 5-fluorouracil and leucovorin) and short-course RT, ypT0 rate was 16.1% and 0.7%, respectively. However, the downstaging effect of short-course RT may also depend on time to surgery [30].

Our study demonstrated poor early outcomes for ypT2 patients in terms of readmission rate and R1 status. Rectal bleeding was the cause of rehospitalization in all cases (10.9% in ypT2 group). This is consistent with other studies describing rectal bleeding as the most frequent complication, with rates ranging from 14% [32] and 27% [33]with TEM and 9% with TAMIS [34]. Our findings indicate that tumors with incomplete pathological response have a higher risk of postoperative bleeding and require careful hemostasis during surgery. Although there may be more bleeding complications in open than closed defects after full-thickness excision [35], we were unable to investigate this issue in our series. Dimensions [36], malignant disease [32] and lateral position of the tumor [37] were identified as risk factors for postoperative bleeding after LE. A correlation with tumor stage was not demonstrated. We hypothesize that there is more tumor cell-induced angiogenesis in tumors invading the muscularis after NT. Alternatively it could be that the macroscopic appearance or the size of the ypT2 tumors could lead to a wider excision of the rectal wall in order to obtain negative margins. The macroscopic appearance or the size of the ypT2 tumors could lead to a wider excision of the rectal wall to obtain negative margins.

Another difference between groups was the R1 resection rate. This was significantly higher in the ypT2 patient group (8.7%). Data from a pooled incidence analysis involving five studies [38] demonstrated a margin positivity rate of 10% (3–24%) after TEM. In the context of ypT2 tumors after LE, our findings are consistent with those of Yang et al. [39] who reported a R1 rate of 12.5%. An R1 resection usually indicates further surgery to reduce the risk of local recurrence. However, no patient with local or distant recurrence had a positive resection margin in our series (Table 3).

We observed health-related quality of life to be unchanged from baseline, with improved emotional well-being in patients treated with LE and NT. This is consistent with that observed after long-term follow up in the CARTS study [18]. Consistent with maintained quality of life in this group is data on anorectal function from the ACOSOG Z6041 [40]. Results confirmed acceptable outcomes after organ-preserving treatment. Outcomes related to quality of life and bowel function are discordant when local excision and radical resection are compared [21, 41] Encouraging results have been obtained from several other studies regarding the benefits of a rectal-preservation strategy [16–19, 22, 42, 43].

Few studies have specifically assessed the outcomes of ypT2 tumors after LE [26, 39]. We reported a 8.7% local recurrence rate in the ypT2 group which is twice the rate reported in ACOSOG Z6041 trial [17] (4.1%). This difference may be related to the inclusion criteria of the ACOSOG study involving only cT2N0 cancers and, we observed a lower local recurrence rate than most of the remaining literature. Unfavorable pathology such as ypT2 correlates with approximately 20% of local recurrence [44]. Several patient or primary tumor-related factors, therapeutic and surgical aspects contribute to local recurrence after NT and LE and should be taken into consideration [44].

Our findings may be used to counsel patients with incomplete response who are not eligible for radical surgery or refuse a permanent stoma or major surgery. However, this study has some limitations. The retrospective nature and potential selection bias make it impossible to draw firm conclusions about NT and LE as definitive treatment for ypT2. More frail patients with a higher CCI and less use of long course RT in the ypT2 group may have had effects on survival and oncological outcomes. Conversely, selection of patients with a good clinical response may have positively affected observed local recurrence rates. Additionally, several variables influencing local recurrence such as tumour grade, as well as lymphovascular and perineural invasion were not considered. Therefore, further prospective studies are warranted, despite the difficulty of randomly assigning patients in the setting of organ-preservation strategies.

## Conclusions

Patients with ypT2 tumors after NT and LE have a higher risk of late-onset rectal bleeding and positive resection margins than patients with complete or near complete response. However, long-term recurrence rates and survival are comparable.
